# Studies in Poor-Law Expenditure

**Published:** 1906-08-18

**Authors:** 


### STUDIES IN POOR-LAW EXPENDITURE.

### PAUPERISM AND POPULATION.

If one were to judge of the wealth and poverty of the different districts of London solely by the proportion of paupers to population, one would make strange blunders. Will it be credited that the Union which shows the highest proportion of pauperism to population, and the highest cost per head of expenditure on paupers per head of population is the Strand? There, according to statistics, the number of paupers is 90.1 per thousand inhabitants, and the cost of maintaining paupers demands £1 11s. 9¾d. from every person in the Union. In the City, paupers cost £1 7s. 11½d. per head of population, and the proportion of paupers is 55.1 per thousand of the population. Any foreigner, reading these figures, would come to the conclusion that these two districts are most appallingly poverty-stricken, instead of being, as they are, very rich. The City of London is, of course, the richest in the whole metropolis, its annual rateable value being £5,019,371, while its estimated population is only 25,523. The Strand has a rateable value of £1,134,094, while its population is only 17,840. But in both of these districts the people who keep up the rateable value, those who earn the largest incomes, live elsewhere, and of the actual day and night residents an exceptionally large proportion are to be reckoned as poor. Our method of census-taking, which deals only with the place where a man spends his nights, and not where he spends his days, is in this way misleading, and the statistics require to be corrected by a certain amount of personal knowledge of the locality. To a certain extent the same rule holds good of the whole of London, and may even be taken to apply to districts at present outside the metropolitan area, though in the latter case the people who live in one parish and work in another are generally poor. But of London, more than of any other great city, must it be said that its inhabitants live two lives, earning their living in one district and spending it in another, and so far as the richer of them are concerned, paying two assessments in consequence. As we have before shown, the two Unions which we have here instanced pay to the receiver of the Metropolitan Common Poor Fund more than they receive back. The City pays annually the enormous sum of £77,391 beyond what it requires for its own needs, and the Strand £7,605. At the opposite pole of proportionate expenditure comes Fulham, where the expenditure on pauperism per head of population is only 2s. 7½d., but this does not really state the actual cost of maintaining the poor of the parish, for Fulham receives from the Common Poor Fund £1,983. Still, Fulham, with Chelsea and Woolwich, may be regarded as being as nearly as possible a self-sustaining parish, the help that each of the three gets from the fund being a comparatively small proportion of its total expenditure.

To form any real basis of comparison, it is necessary to take parishes which are fairly equal in the number and character of their population. Such, for example, are Bermondsey and Bethnal Green. Their population is almost exactly equal—Bermondsey 129,856, and Bethnal Green 129,118. The latter is, according to the rateable value, the poorer of the two, being assessed at £527,941, while the value of Bermondsey is £925,000. Both are inhabited by poor people, and one would expect that the proportion of paupers to population would be fairly equal. But the paupers in Bermondsey number 6,052, of whom 3,164 are indoor and 2,888 outdoor; while in Bethnal Green the total pauperism is 3,082, of whom 2,603 are indoor and 479 outdoor. The proportions in the two districts are 46.6 per thousand in Bermondsey, as against 23.9 in Bethnal Green. In these two cases the difference in numbers can come only from a difference in administration, and it will be seen that the greatest disproportion is in the number of outdoor poor. As the Rev. P. S. G. Propert, Chairman of the Fulham Guardians, pointed out when laying the annual report before his Board, the lavish giving of out-relief never seems to lessen the number of the inmates of the workhouse, and this is a moral which seems to be pointed by the case of Bermondsey.

A similar inference may be drawn from Holborn, where the indoor paupers number 3,699 and the outdoor ones 2,009; from Greenwich, where an indoor pauperism of 2,812 is nearly balanced by an outdoor pauper population of 2,674; from Hackney, where the indoor paupers number 3,287 and outdoor 2,744; from Lambeth, indoor 3,580, outdoor 2,682; from Lewisham, indoor 1,229, and outdoor 1,682; from Mile End, indoor 1,772, and outdoor 1,383; and from Wandsworth, indoor 3,615, and outdoor 2,358. It is hardly contradicted by the fact that in Poplar and Camberwell the outdoor paupers outnumber the indoor ones. Thus in Poplar the numbers are, indoor 3,568, and outdoor 4,165; while Camberwell shows something more amazing still—indoor 2,735 and outdoor 4,593. Camberwell is a much richer Union than Poplar, its valuation being £1,293,905, as against Poplar's £815,529; and the number of paupers is less in proportion to population—Camberwell, 27.8 per thousand, and Poplar, 45.7—so that it does not feel the burden of the rates so severely as Poplar does; but having these figures before us, we would suggest that when the affairs of Poplar have been investigated, those of Camberwell should receive attention. In fact, it may be set down as an axiom that there may be many indoor paupers where there are but few outdoor ones; but that where there are many outdoor paupers indoor ones will be numerous also. Nowhere in London do we find a relatively or absolutely large number of outdoor paupers in the same place as a relatively or absolutely small number of indoor ones.

But the converse is not equally true. Taking the three neighbouring parishes of Stepney, Whitechapel, and Mile End—the district which is commonly called the "East End," and to which popular sentiment mistakenly believes all the poverty of London to be confined—and we find that Mile End has, as has been said, something approaching an equal number of indoor and outdoor paupers—1,772 of the former and 1,383 of the latter. Stepney has nearly as many people in its workhouse and kindred institutions—1,602—but its outdoor paupers number only 510; while in Whitechapel, though the indoor paupers number 1,587, the outdoor recipients of relief are only 62. One can hardly help drawing the inference that indoor pauperism is an inevitable condition of our present social conditions and of the weakness of human nature; but outdoor pauperism is largely a manufactured article. Or to put it otherwise, indoor pauperism is a civic misfortune; outdoor pauperism an administrative crime—or, as the epigrammatist said, worse than a crime, a blunder.

